# Psychometric properties the Iranian version of Older People’s Quality Of Life questionnaire (OPQOL)

**DOI:** 10.1186/s12955-018-1002-z

**Published:** 2018-09-05

**Authors:** Maryam Nikkhah, Majideh Heravi-Karimooi, Ali Montazeri, Nahid Rejeh, Hamid Sharif Nia

**Affiliations:** 1 Shahed University, Faculty of Nursing & Midwifery, Tehran, Iran; 2 Elderly Care Research Center, Shahed University, Faculty of Nursing & Midwifery, Opposite Holy Shrine of Imam Khomeini-Khalij Fars Expressway, Tehran, Iran; 3 Population Health Research Group, Health Metrics Research Center, Iranian Institute for Health Sciences Research, ACECR, Tehran, Iran; 4 Amol Faculty of Nursing and Midwifery, Mazandaran University of Medical Sciences, Sari, Iran

**Keywords:** Validity, Reliability, Quality of life, Older, The older People’s quality of life questionnaire (OPQOL)

## Abstract

**Background:**

Assessing quality of life (QOL) in elderly needs specific instruments. The Older People’s Quality of Life Questionnaire (OPQOL-35) is one of the common tools that used for measuring quality of life in elderly populations. The questionnaires contains 35 items tapping into eight domains including life overall, health, social relationships and participation, independence, control over life and freedom, home and neighborhood, psychological and emotional well-being, financial circumstances, culture and religion. This study aimed to translate and validate the OPQOL-35 in Iran.

**Methods:**

Forward-backward procedure was applied to translate the original questionnaire from English into Persian. Then following qualitative face and content validity, a sample of elderly people completed the questionnaire. In order to evaluate the construct validity, exploratory and confirmatory factor analyses was performed. Subsequently, convergent and divergent validity of the factors were evaluated. Reliability was evaluated by performing internal consistency analysis and Intraclass Correlation Coefficients (ICC).

**Results:**

In all 500 older people completed the questionnaire. The mean age of participant was 68.92 (SD = 6.97) years, and mostly were males (66.6%). The result of exploratory factor analysis showed 8 factors with Eigen values of greater than one, which explained 67.4% of the variance observed. Confirmatory factor analysis showed acceptable fit indexes for the data [Comparative Fit Index (CFI) = 0.92, Minimum Discrepancy Function by Degrees of Freedom divided (CMIN/DF) = 2.832, Root Mean Square Error of Approximation (RMSEA) = 0.067]. The convergent and divergent validity did not support three latent factors (Life overall, Independence, control over life, freedom and Psychological and emotional well-being). Convergent and divergent validity shown that construct fulfilled for the health, social relationships and participation, home and neighborhood, financial circumstances, culture and religion latent factors, however the results did not support the convergent and divergent validity for three latent factors (Life overall, Independence, control over life, freedom and Psychological and emotional well-being). Cronbach’s alpha coefficient for the subscales ranged from 0.65–0.95. Test-retest reliability (ICC) of the questionnaire with two weeks interval were ranged from 0.88–0.95 indicating a good range of reliability.

**Conclusion:**

The findings suggest that the Iranian version of OPQOL-35 is a valid measure for assessing quality of life in elderly populations in different settings.

**Electronic supplementary material:**

The online version of this article (10.1186/s12955-018-1002-z) contains supplementary material, which is available to authorized users.

## Background

The world population is aging [1]. An aging population can be seen, not only in developed countries but also in developing countries [2]. Also recent reports predicted that within 10 years, the elderly population over 65 years will increase from 7 to 14% by 2040 [3]. The aging of population is often considered as a global public health success, although at the same time it is widely acknowledged as a public health challenge in high-income countries [4, 5]. According to the 2011 census the population of elderly people accounted for 8.2% in Iran. Also predicted that this rate will increase to 22% in 2046 [6, 7]. Thus, it is argued that if aging is a challenge for developed countries, therefore in the lower and middle-income countries such as Iran it is a more significant challenge [4, 5].

Quality of life has become an end point in the evaluation of multi sector public policy, including health, social, community and environmental policies [8]. Quality of life has long been used to evaluate various range of health and social care interventions [9]. Quality of Life is a multidimensional concept and is defined as ‘individuals’ perceptions of their position in life in the context of the culture and value system in which they live, and in relationship to their goals, expectations and standard’ [10]. We are witnessing a significant prolongation of life expectancy, but maintaining high quality of life is important. [11]. In fact, Active aging is defined as ‘the process of optimizing the opportunities for physical, cognitive, and social health all over life with the aim of increasing a healthy life expectancy, efficiency and quality of life in older age’ which is more important than an increase in the aging population [2, 12]. Thus, direct or indirect effects of age on quality of life led researchers in geriatrics interested in determining the factors that are related to the quality of life in older people [2, 13, 14].

Since Health-Related Quality Of Life (HRQOL) of elderly people is becoming an important issue of public health, measuring quality of life of the elderly can be helpful for health program planning in future [1]. So far, there are a large number of instruments for measuring quality of life. They are different widely in their conception, construct and content [15]. Studies have shown that from 1970 instruments for measuring quality of life exponentially increased [13]. There are several generic quality of life measures for elderly people including the Older People’s Quality of Life Questionnaire (CASP-19), the World Health Organization Quality of Life – OLD (WHOQOL-OLD) and the World Health Organization Quality of Life – AGE (WHOQOL-AGE) [16–18]. One of the new instruments available to measure quality of life of elderly people is the Older People’s Quality of Life Questionnaire (OPQOL-35) [19]. This instrument assesses quality of life in older people that can provide more information about quality of life in this population. In addition, this questionnaire has been shown recently not only excellent use in healthy people but can be used in old patients with mild or moderate dementia [20]. This tool has been translated into various languages and has been used in several countries, including Britain [21], Australia [19], China [22], Albania [23], and India [24]. The aim of this study was to evaluate the psychometric properties of the Persian version of OPQOL-35.

## Methods

### The questionnaire

The Older People Quality of Life consists of 35 statements, each statement has 5 repose categories (strongly disagree’, ‘disagree’, ‘neither agree nor disagree’, ‘agree’ and ‘strongly agree’, with a score ranging from of 1 to 5). Higher scores represent better quality of life. The total score range from 35 (worst possible QOL) to 175 (best possible QOL). The full OPQOL questionnaire cover: life overall (4 items, score range 4–20), health (4 items, score range 4–20), social relationships and participation (8 items, score range 8–40), independence, control over life and freedom (5 items, score range 5–25), home and neighborhood (4 items, score range 4–20), psychological and emotional well-being (4 items, score range 4–20), financial circumstances (4 items, score range 4–20), culture and religion (2 items, score range 2–10) [5].

### Translation

After asking for the author’s permission, two skilled Iranian professionals (doctoral nurses) translated the OPQOL-35 from English into Persian. The two Persian translations were compared and combined to create a consolidated forward translation. This version then was backward translated into English by two medical specialists. Two specialists who had no previous knowledge of the QOL were compared the backward translation with the original questionnaire to ensure that the main concepts were transferred into the Persian version. Then cognitive interviewing was used to assess comprehension and then refine these based on participants’ feedbacks.

Consequently we performed face and content validity as described in the following sections:

1. Content Validity: quantitative content validity was performed by calculating Content Validity Ratio (CVR) and Content Validity Index (CVI). CVR reflects whether the items were deemed essential by professionals (2 doctoral nurses, 2 geriatric nursing professionals as faculty of members of two medical sciences universities in Iran and 2 clinical nurse with the experience of working by older people). Accordingly, five experts were asked to rate the essentiality of the OPQOL-35 items on a three-point scale as follows: Not essential: 1; Useful but not essential: 2; and Essential: 3 [25]. On the other hand, CVI indicates the degree to which the items of scale are simple, accurate, and clear. Thus, we asked the five board members to evaluate the simplicity, relevance and clarity of the OPQOL-35 items on a four-point Likert scale. In addition we performed qualitative content validity. In doing so we asked five experts to evaluate the phrase, item allocation, and scaling of the items [26].
2. Face validity: we used both qualitative and quantitative approaches. Qualitative face validity involved ten older people who evaluated the questionnaire for difficulty, relevance, and ambiguity. Quantitative face validity assessment was assessed using the item impact technique. The same ten older people who assisted with the qualitative review were asked to evaluate the importance of the items on a 5-point Likert scale ranging from 1 (Not important) to 5 (Completely important). An impact score for each item was calculated by importance frequency. If the impact score of the item was more than 1.5, the item was considered appropriate [27, 28].

### Participants

A methodological study with quantitative approach was conducted and a sample of old people living in Tehran was entered into the study in 2016. The inclusion criteria for participant selection included: 1) Age over 60 years; 2) necessary communication skills; and 3) the absence of psychological problems, cognitive impairment and hearing loss. Munro (2005) states that the required number of respondents for Exploratory Factor Analysis (EFA) is between 3 and 10 participants per item, or a total of 100 to 200 respondents [29]. As the samples of this study, 500 older people were recruited from elderly centers of Tehran Municipality.

### Statistical analysis

Ceiling and floor effects were appraised using percentage of scores at the boundaries of the scaling range (e.g. 0 and 100). Floor or ceiling effects are matter of concern if more than 15% of respondents achieve the lowest or highest possible score, respectively [30]. Construct validity was tested by several tests. The factor structure of the OPQOL-35 was evaluated by performing exploratory factor analysis with varimax rotation [31]. The Kaiser–Meyer–Olkin (KMO) and Bartlett’s test of sphericity also were performed. Kaiser indicates values between 0 and 0.49 as unacceptable, 0.5–0.7 as mediate; 0.7–0.8 as good, 0.8–0.9 as great, and > 0.9 as excellent [32]. The number of factors extracted was based on eigenvalues and the scree plot. The scree plot is a heuristic graphic method that consists of: the eigenvalues (y-axis) against the components (x-axis), and inspecting the shape of the resulting curve in order to detect the point at which the curve changes significantly. This point on the curve indicates the maximum number of components to retain [33]. Eigenvalues greater than one, and factor loadings greater than 0.4 were the criteria used to select the factors [34, 35]. Missing values were replaced with the mean. The exploratory factor analysis was essential for the questionnaire since the authors in one study reported 8 factors and in another study reported 9 factors [8, 36]. Then by performing Confirmatory Factor Analysis (CFA) and using model fit indexes such as Chi-square (χ2), Chi-square/degree of freedom ratio, Comparative Fit Index (CFI), Incremental Fit Index (IFI), and Root Mean Square Error of Approximation (RMSEA) the obtained factor structure was confirmed [37]. The range of acceptable fit indexes for confirmatory factor analysis is presented in Table 1. Subsequently, convergent and divergent validity of the factors were evaluated by measuring Average Variance Extracted (AVE), Maximum Shared Squared Variance (MSV) and Average Shared Squared Variance (ASV) [37]. To determine convergent validity, the AVE of the constructs should be more than 0.50. For divergent validity, both MSV and ASV should be less than AVE [37, 38]. EFA was analyzed using SPSS-22. CFA was performed using the Analysis of Moment Structure (AMOS) software version 21. The reliability of OPQOL was assessed through evaluating its internal consistency calculating Cronbach’s alpha (α) for each domain [37, 39, 40]. Cronbach’s α coefficient of 0.7 or above was considered satisfactory [41]. Also, to examine the test–retest reliability of the OPQOL, a sub-sample of older people (*n* = 70) completed the questionnaire once again in a 2-week interval ensuring that their health did not change between the two assessments. The test-retest reliability of the recall ratings was evaluated using Intraclass Correlation Coefficients (ICC) for absolute agreement at the level of individual items where the ICC of more than 0.8 was considered acceptable [41–43].

**Table 1:** The range of acceptable fit indexes of confirmatory factor analysis [44]

Indexes	Acceptable range
χ^2^ *p*-value (Chi-squared *p*-value)	> 0.05
RMSEA (Root Mean Square Error of Approximation)	Good 0.08>, medium 0.1–0.08 and poor 0.1<
Comparative Fit Index (CFI)	≥0.9
PNFI(Parsimonious Normed Fit Index)	> 0.5
PCFI(Parsimonious Comparative Fit Index)	> 0.5
AGFI(Adjusted Goodness of Fit Index)	> 0.8
CMIN/DF (Minimum Discrepancy Function by Degrees of Freedom divided)	Good 3 > and 5 > acceptable

### Ethics

The ethics committee of Shahed University confirmed the study. The goals and procedures of the research were explained to all participants. They were confident of confidentiality and that they could be excluded from the study at any time. Each participant initially wrote informed consent and then completing the instrument. Completed instruments were stored securely.

## Results

### The study sample

In all 500 elderly participated in the study. Of these, 333 (66.6%) were men. In addition, 400 (80%) were married, 208 (41.6%) were in the age range 60–65 years, 305 (61%) were retired, 399 (79.8%) lived with spouse, and 417 (83.4) lived in a private house. The highest mean score was for physical functional dimension 82.38 (SD = 15.27) and the lowest mean score was for public health dimension 50.07 (SD = 15.57). No floor or ceiling effects were observed for the total score of Persian OPQOL. Table 2 shows the demographic characteristics of the study sample.

**Table 2:** Demographic Characteristics of the study sample (*n* = 500)

	Number	%
Gender		
Male	333	66.6
Female	167	33.4
Age group (years)		
60–65	208	41.6
66–71	127	25.4
72–77	95	19
78–83	54	10.8
84–90	16	3.2
Marital status		
Married	400	80
Single	2	0.4
Widow	88	17.6
Divorced	6	1.2
Truce	4	0.8
Employment status		
Housewife	138	27.6
Practitioner	22	4..4
Retired	305	61
Unable	35	7
Living conditions		
Alone	59	11.8
With spouse	399	79.8
With Children	41	8.2
With Others	1	0.2
Housing		
Private house	417	83.4
Impersonal house	83	16.6
Financial status		
Not enough	27	5.4
At least	183	36.6
Moderate	248	49.6
Enough	42	8.4
Educational level		
Illiterate	65	13
Reading and writing	53	10.6
Elementary	186	37.2
Diploma	124	24.8
University education	72	14.4

### Psychometric properties

**Fig. 1:**
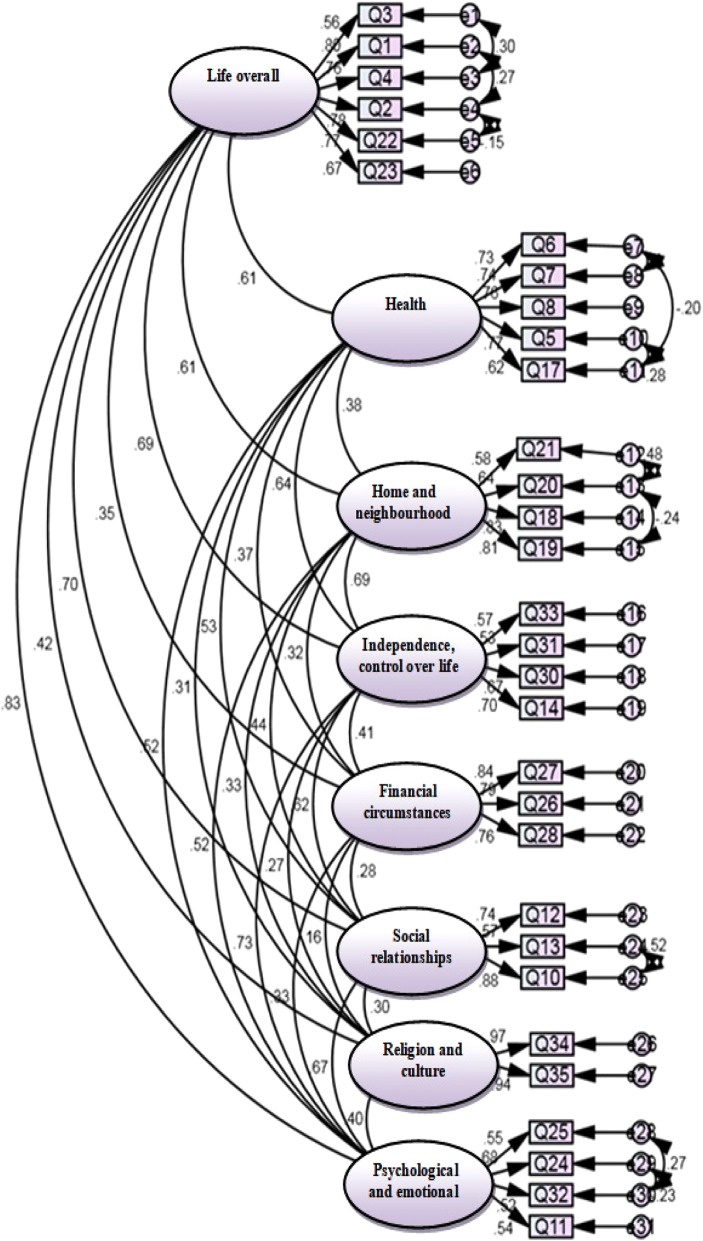
The results obtained from confirmatory factor analysis for the OPQOL-35

1. Exploratory factor analysis: An exploratory factor analysis with varimax rotation was used to assess the construct validity of the questionnaire. The KMO was 0.897 supporting that the sampling was adequate and the Bartlett’s test of sphericity was significant (496, *p* < 0.001) indicating that the variables in the questionnaire were related and that a CFA was thus useful. Eight factors were extracted and identified using a minimum eigenvalue of 1 as the factor criterion. The eight factors accounted for 67.4% of the variance observed. The results are shown in Table 3. However, additional item-domain correlation matrix also was provided to ensure that the instrument has a good construct (see Additional file 1).
2. Confirmatory factor analysis: the results obtained from the confirmatory factory analysis indicated a good fit for data as follows: CFI = 0.92, PCFI = 0.734, PNFI = 0.701, CMIN/DF = 2.832, RMSEA = 0.067, AGFI = 0.831, GFI = 0.87 were confirmed goodness of fit of final model (Fig. 1). The range of acceptable fit indexes of confirmatory factor analysis are presented in Table 1.
3. Convergent validity, divergent validity: Table 4 shows AVE for all factors. Except for following factors (Independence, control over life, freedom and Psychological and emotional well-being) was greater than 0.5, indicating acceptable convergent validity. On the other hand MSV and ASV for all factors except for Life overall, Independence, control over life, freedom and Psychological and emotional well-being was less than AVE, indicating a good divergent validity.
4. Reliability: Table 5 shows the instrument had a high internal consistency (Cronbach’s α = 0.65–0.95). The ICC was 0.95 (95% CI: 0.93–0.97, *p* < 0.001) representing appropriate sustainability for the questionnaire. Cronbach’s alpha coefficients if item deleted are not shown but is available as supplementary information (see Additional file 2).

**Table 3:** Factor structure of the OPQOL derived from principal component analysis

Factor	Item (item number)	Factor loading	Eigenvalue	% variance observed
*Life overall*			3.70	11.56
	I look forward to things (3)	0.764		
	I enjoy my life overall (1)	0.746		
	Life gets me down (4)	0.744		
	I am happy much of the time (2)	0.707		
	I take life as it comes and make the best of things (22)	0.491		
	I feel lucky compared to most people (23)	0.458		
*Health*			3.44	10.76
	Pain affects my well-being (6)	0.829		
	My health restricts me looking after myself or my home (7)	0.800		
	I am healthy enough to get out and about (8)	0.754		
	I have a lot of physical energy (5)	0.751		
	I have a lot of control over the important things in my life (17)	0.426		
*Home and neighbourhood*			2.69	8.43
	I find my neighbourhood friendly (21)	0.846		
	I get pleasure from my home (20)	0.775		
	I feel safe where I live (18)	0.647		
	The local shops, services and facilities are good overall (19)	0.628		
*Independence, control over life, freedom*			2.62	8.19
	I have responsibilities to others that restrict my social or leisure activities (33)	0.656		
	I try to stay involved with things (31)	0.620		
	I have social or leisure activities/hobbies that I enjoy doing (30)	0.615		
	I am healthy enough to have my independence (14)	0.539		
*Financial circumstances*			2.49	7.78
	I have enough money to pay for household repairs or help needed in the house (27)	0.866		
	I have enough money to pay for household bills (26)	0.846		
	I can afford to buy what I want to (28)	0.766		
*Social relationship*			2.35	7.37
	I’d like more people to enjoy life with (12)	0.760		
	I have my children around which is important (13)	0.676		
	I would like more companionship or contact with other people (10)	0.617		
*Religion/culture*			2.21	6.93
	Religion, belief or philosophy is important to my quality of life (34)	0.919		
	Cultural/religious events/festivals are important to my quality of life (35)	0.916		
*Psychological and emotional well-being*			2.05	6.42
	If my health limits social/ leisure activities, then I will compensate and find something else I can do (25)	0.743		
	I tend to look on the bright side (24)	0.547		
	I do paid or unpaid work or activities that give me a role in life (32)	0.544		
	I have someone who gives me love and affection (11)	0.413

**Table 4:** Convergent validity, divergent validity and construct reliability of OPQOL

Dimension	AVE	MSV	ASV
Life overall	0.53	0.681	0.384
Health	0.528	0.403	0.245
Home and neighborhood	0.519	0.471	0.238
Independence, control over life, freedom	0.388	0.527	0.357
Financial circumstances	0.635	0.171	0.106
Social relationships	0.548	0.487	0.281
Religion/culture	0.907	0.173	0.103
Psychological and emotional well-being	0.331	0.681	0.353

*AVE* Average Variance Extracted, *MSV* Maximum Shared Squared Variance, *ASV* Average Shared Squared Variance

**Table 5:** Cronbach’s Alpha and Intra class correlation index of OPQOL

Dimension	Cronbach’s α	ICC (*n* = 70)	95% CI
Life overall	0.87	0.944	0.90–0.96
Health	0.83	0.956	0.93–0.97
Social relationships	0.72	0.945	0.91–0.96
Independence, control over life, freedom	0.71	0.899	0.83–0.93
Home and neighbourhood	0.82	0.894	0.83–0.93
Psychological and emotional well-being	0.653	0.89	0.82–0.93
Financial circumstances	0.834	0.944	0.91–0.96
Religion/culture	0.951	0.889	0.82–0.93
Total	0.92	0.92	0.91–0.93

## Discussion

The purpose of this study was to translate, conduct reliability and validity testing of the OPQOL in older people of Iran. Most of geriatric studies aimed to investigate the QOL in older people. However, an absolute prerequisite of these studies is the availability of a standard, valid and reliable questionnaire. The study findings revealed that the OPQOL is a multidimensional scale and has acceptable psychometric properties.

Using exploratory factor analysis the results demonstrated that the questionnaire contained eight factors that jointly explained 67.4% of the total variance observed. Factors 1 and 2 (life overall and health) contributed the most to the total variance 11.5 and 10.7, respectively. Accordingly, the greatest contribution of these two factors to the total variance is perfectly justifiable. Perhaps contribution of overall living conditions of older people and their concern about health are universal factors that might contribute to quality of life in this population. So, OPQOL is a multidimensional scale in older people. However, comparing factor structure of the OPQOL in different settings showed interesting results. Bowling (2010) evaluated the psychometric properties of the OPQOL in 3 groups of older people and reported the presence of 9 factors which explained cumulatively 60.5% of the total variance in QOL between respondents; where component 1 explained the largest proportion (24.0%) of the variance observed [36]. Chen et al. (2014) evaluated the psychometric properties of the OPQOL in a sample of 618 older people living alone in China and reported 8 factors labelled ‘leisure and social activities’, ‘psychological well-being’, ‘health and independence’, ‘financial circumstances’, ‘social relationships’, ‘home and neighbourhood’, ‘culture/religion’ and ‘safety’, accounting for 63.7% of the variance. They reported that the factor structure was similar to the original version but differences were also found [22]. The results of factor analysis suggest that the Persian version of the eight-factor structure of the OPQOL is consistent with that conducted by Bowling et al. (2010) and Chen et al. (2014). Consequently, the eight factors OPQOL has acceptable construct validity.

This study validated this scale by the use of CFA. CFA can evaluate goodness of fit results of factor structure of a scale, which can provide more precise and conclusive evaluation of latent factors [44]. In the present study, data analysis confirmed that the final model was a good fit. The present study used AVE, MSV, and ASV for assessment of convergent and divergent validity. Convergent and divergent validity show satisfactory results for the following factors: health, social relationships and participation, home and neighborhood, financial circumstances, culture and religion. However the results did not support the convergent and divergent validity for three latent factors (Life overall, Independence, control over life, freedom and Psychological and emotional well-being). This result might be due to the low loading of one of the items in three factors. Moreover, high measurement errors may be another explanation for such observation. The measurement model did not show how measurement items logically and systematically contributed to the latent constructs [45].

The study showed that the instrument’s reliability was excellent. More importantly, when the Cronbach’ alpha coefficients were calculated separately for the eight factors, high values for Cronbach’s alpha showed that good internal consistency exists for eight factors. The total Cronbach’s alpha of the Persian OPQOL was 0.92. The factor of ‘psychological and emotional well-being’ had a Coronbach’s alpha coefficient less than 0.70. Although the Cronbach’s alpha coefficient is enhanced by a large number of items, the high Cronbach’s alpha coefficient observed for the entire measure cannot be interpreted as indicating a unidimensional measure [44]. Similarly studies reported good internal consistency for the OPQOL in different settings. Rathnayake et al. evaluated the quality of life and Its determinants among older people living in the rural community in Sri Lanka and reported 0.862 internal consistency coefficients [46]. Also, Bilotta et al. reported 0.78 internal consistency coefficients for the Italian outpatient population [21]. Furthermore, Bowling et al. (2011) found 0.9 Cronbach’s alpha coefficients in their research [8]. Bazaadut evaluated the relationship between caregiver psychosocial factors and the quality of life of elderly at home in a sample of 400 older people living the Tamale township and reported 0.81 for internal consistency coefficients [47]. In the study by Chen et al., who had calculated between 0.7–0.97 Cronbach’s alpha coefficients [22].

The test-retest ICC of the OPQOL was between 0.88–0.95. The lowest ICC coefficient was related to the ‘psychological and emotional well-being’ subscale. According to Houser (2013), stability values of greater than 0.7 are considered as satisfactory. Test-retest is one of the common reliability assessment methods that assess the stability and the repeatability of an instrument. Polit et al. (2012) considered stability values of greater than 0.7, 0.8, and 0.9 as satisfactory, very good, and ideal, respectively [48]. Accordingly, the Persian OPQOL showed very good stability. Bowling et al. (2011) reported between 0.4–0.78 test–retest [8] and Chen et al. (2014) found 0.53–0.87 test-retest ICC in their research [22]. Compared to the original version, the Persian version had better results with regard to the domains.

One of the integral needs of each community is to pay more attention to the elderly. As suggested identification of factors affecting on the quality of life may facilitate the design of interventions for the elderly [23]. In fact as indicated by Bowling et al. OPQOL could have prognostic value in research on older people [5]. Similarly our findings provide support to the prognostic value of QOL measures in older people. Thus, the OPQOL-35 might be superior to other broader measures of QOL in older age.

The present study has certain strengths and limitations. Inclusion of a teams of experts (translators, statistician, and methodologists) in designing the study and evaluating the questionnaire are the strengths. However, issues related to our sample might limit the findings. For instance we did not examined the sample for the emotional health. Thus this might have had impact on the study findings. In addition we did not collect data on clinical variable for testing criterion validity. This also might limit the findings. Finally as suggested convergent and divergent validity might be better tested by using another pre-validated quality of life questionnaire for the elderly (such as WHOQOL-OLDD etc).

## Conclusion

The findings suggest that the Persian version of Older People Quality of Life (OPQOL) instrument is a reliable and valid measure of quality of life in this population and now can be used in geriatrics outcome studies. The OPQOL is easy to understand and takes less than 15 min to be completed. The OPQOL is used for measuring QOL and assessing the various range of health and social care interventions in older people.

## Additional files

Additional file 1: Item-domain correlation matrix for the OPQOL-35. (DOC 185 kb)

Additional file 2: Cronbach’s alpha coefficients for the OPQOl-35 if item deleted. (DOC 92 kb)

## Abbreviations

AGFI: Adjusted Goodness of Fit Index
AMOS: Analysis of Moment Structure
ASV: Average Shared Squared Variance
AVE: Average Variance Extracted
CASP-19: Older People’s Quality of Life Questionnaire
CFA: Confirmatory Factor Analysis
CFI: Comparative Fit Index
CMIN/DF: Minimum Discrepancy Function by Degrees of Freedom divided
CVI: Content Validity Index
CVR: Content Validity Ratio
EFA: Exploratory Factor Analysis
HRQOL: Health-Related Quality Of Life
ICC: Intraclass Correlation Coefficients
IFI: Incremental Fit Index
KMO: Kaiser–Meyer–Olkin
MSV: Maximum Shared Squared Variance
OPQOL-35: Older People’s Quality of Life Questionnaire
PCA: Principal Component Analysis
PCFI: Parsimonious Comparative Fit Index
PNFI: Parsimonious Normed Fit Index
QOL: Quality Of Life
RMSEA: Root Mean Square Error of Approximation
WHOQOL-AGE: World Health Organization Quality of Life – AGE
WHOQOL-OLD: World Health Organization Quality of Life – OLD
