# Supplementary material for: Psychometric properties the Iranian version of Older People’s Quality Of Life questionnaire (OPQOL)
Source: Health Qual Life Outcomes. 2018 Sep 5;16:174. doi: 10.1186/s12955-018-1002-z (PMC6126014; doi:10.1186/s12955-018-1002-z)
Supplement: Supplementary file 1 — Item-domain correlation matrix for the OPQOL-35. (DOC 185 kb) [file 12955_2018_1002_MOESM1_ESM.doc]

**Item-domain correlation matrix for the OPQOL-35‎**

|  | **Life overall** | **Health** | **Social relationships** | **Independence, control over life, freedom** | **Psychological and emotional well-being** | **Financial circumstances** | **Home and neighbourhood** | **Religion/ culture** |
| --- | --- | --- | --- | --- | --- | --- | --- | --- |
| I enjoy my life overall | .860^**^ | .420^**^ | .537^**^ | .506^**^ | .618^**^ | .288^**^ | .447^**^ | .355^**^ |
| I am happy much of the time | .838^**^ | .371^**^ | .559^**^ | .486^**^ | .576^**^ | .283^**^ | .425^**^ | .321^**^ |
| I look forward to things | .781^**^ | .269^**^ | .354^**^ | .391^**^ | .411^**^ | .207^**^ | .279^**^ | .167^**^ |
| Life gets me down | .871^**^ | .406^**^ | .521^**^ | .507^**^ | .599^**^ | .258^**^ | .372^**^ | .284^**^ |
| I have a lot of physical energy | .396^**^ | .836^**^ | .402^**^ | .416^**^ | .406^**^ | .218^**^ | .273^**^ | .223^**^ |
| Pain affects my well-being | .330^**^ | .859^**^ | .325^**^ | .337^**^ | .352^**^ | .139^**^ | .173^**^ | .173^**^ |
| My health restricts me looking after myself or my home | .379^**^ | .852^**^ | .385^**^ | .439^**^ | .350^**^ | .253^**^ | .191^**^ | .206^**^ |
| I am healthy enough to get out and about | .362^**^ | .795^**^ | .429^**^ | .496^**^ | .364^**^ | .218^**^ | .335^**^ | .232^**^ |
| My family, friends or neighbours would help me if needed | .334^**^ | .278^**^ | .534^**^ | .278^**^ | .402^**^ | .292^**^ | .267^**^ | .257^**^ |
| I would like more companionship or contact with other people | .503^**^ | .407^**^ | .731^**^ | .554^**^ | .492^**^ | .240^**^ | .379^**^ | .218^**^ |
| I have someone who gives me love and affection | .332^**^ | .322^**^ | .644^**^ | .302^**^ | .464^**^ | .225^**^ | .231^**^ | .357^**^ |
| I’d like more people to enjoy life with | .436^**^ | .281^**^ | .662^**^ | .365^**^ | .476^**^ | .140^**^ | .310^**^ | .256^**^ |
| I have my children around which is important | .341^**^ | .179^**^ | .491^**^ | .247^**^ | .377^**^ | .143^**^ | .228^**^ | .243^**^ |
| I am healthy enough to have my independence | .410^**^ | .457^**^ | .489^**^ | .754^**^ | .394^**^ | .344^**^ | .416^**^ | .171^**^ |
| I can please myself what I do | .416^**^ | .368^**^ | .446^**^ | .625^**^ | .377^**^ | .279^**^ | .304^**^ | .139^**^ |
| The cost of things compared to my pension/ income restricts my life | .391^**^ | .275^**^ | .307^**^ | .669^**^ | .239^**^ | .347^**^ | .213^**^ | .099^*^ |
| I have a lot of control over the important things in my life | .363^**^ | .441^**^ | .470^**^ | .654^**^ | .405^**^ | .335^**^ | .348^**^ | .230^**^ |
| I feel safe where I live | .435^**^ | .225^**^ | .427^**^ | .412^**^ | .393^**^ | .260^**^ | .827^**^ | .312^**^ |
| The local shops, services and facilities are good overall | .406^**^ | .191^**^ | .428^**^ | .423^**^ | .359^**^ | .227^**^ | .824^**^ | .223^**^ |
| I get pleasure from my home | .365^**^ | .309^**^ | .354^**^ | .403^**^ | .381^**^ | .277^**^ | .776^**^ | .209^**^ |
| I find my neighbourhood friendly | .249^**^ | .203^**^ | .329^**^ | .345^**^ | .345^**^ | .270^**^ | .804^**^ | .183^**^ |
| I take life as it comes and make the best of things | .616^**^ | .400^**^ | .598^**^ | .507^**^ | .791^**^ | .222^**^ | .446^**^ | .316^**^ |
| I feel lucky compared to most people | .554^**^ | .404^**^ | .511^**^ | .331^**^ | .766^**^ | .161^**^ | .339^**^ | .280^**^ |
| I tend to look on the bright side | .468^**^ | .265^**^ | .490^**^ | .333^**^ | .773^**^ | .227^**^ | .306^**^ | .205^**^ |
| If my health limits social/ leisure activities, then I will compensate and find something else I can do | .364^**^ | .265^**^ | .490^**^ | .319^**^ | .711^**^ | .069 | .291^**^ | .166^**^ |
| I have enough money to pay for household bills | .248^**^ | .267^**^ | .244^**^ | .334^**^ | .261^**^ | .793^**^ | .230^**^ | .113^*^ |
| I have enough money to pay for household repairs or help needed in the house | .236^**^ | .235^**^ | .261^**^ | .337^**^ | .178^**^ | .847^**^ | .202^**^ | .132^**^ |
| I can afford to buy what I want to | .308^**^ | .189^**^ | .309^**^ | .476^**^ | .208^**^ | .847^**^ | .379^**^ | .121^**^ |
| I cannot afford to do things I would enjoy | .155^**^ | .067 | .146^**^ | .249^**^ | .039 | .596^**^ | .163^**^ | .085 |
| I have social or leisure activities/hobbies that I enjoy doing | .409^**^ | .301^**^ | .622^**^ | .532^**^ | .412^**^ | .263^**^ | .382^**^ | .186^**^ |
| I try to stay involved with things | .258^**^ | .262^**^ | .622^**^ | .395^**^ | .360^**^ | .168^**^ | .266^**^ | .185^**^ |
| I do paid or unpaid work or activities that give me disagree a role in life | .317^**^ | .205^**^ | .629^**^ | .363^**^ | .429^**^ | .070 | .299^**^ | .217^**^ |
| I have responsibilities to others that restrict my social or leisure activities | .367^**^ | .183^**^ | .442^**^ | .719^**^ | .298^**^ | .251^**^ | .401^**^ | .123^**^ |
| Religion, belief or philosophy is important to my quality of life | .320^**^ | .252^**^ | .368^**^ | .215^**^ | .315^**^ | .163^**^ | .288^**^ | .977^**^ |
| Cultural/religious events/festivals are important to my quality of life | .332^**^ | .229^**^ | .385^**^ | .212^**^ | .306^**^ | .123^**^ | .273^**^ | .976^**^ |

* Significant at the 0.05 level.

** Significant at the 0.01 level.
