# Supplementary material for: Psychometric properties the Iranian version of Older People’s Quality Of Life questionnaire (OPQOL)
Source: Health Qual Life Outcomes. 2018 Sep 5;16:174. doi: 10.1186/s12955-018-1002-z (PMC6126014; doi:10.1186/s12955-018-1002-z)
Supplement: Supplementary file 2 — Cronbach’s alpha coefficients for the OPQOl-35 if item deleted. (DOC 92 kb) [file 12955_2018_1002_MOESM2_ESM.doc]

**Cronbach’s alpha coefficients for the OPQOL-35 if item deleted**

| **Item** | **Cronbach's Alpha if Item Deleted** |
| --- | --- |
| I enjoy my life overall | .923 |
| I am happy much of the time | .923 |
| I look forward to things | .925 |
| Life gets me down | .923 |
| I have a lot of physical energy | .924 |
| Pain affects my well-being | .926 |
| My health restricts me looking after myself or my home | .925 |
| I am healthy enough to get out and about | .925 |
| My family, friends or neighbours would help me if needed | .925 |
| I would like more companionship or contact with other people | .923 |
| I have someone who gives me love and affection | .925 |
| I’d like more people to enjoy life with | .925 |
| I have my children around which is important | .926 |
| I am healthy enough to have my independence | .924 |
| I can please myself what I do | .925 |
| The cost of things compared to my pension/ income restricts my life | .926 |
| I have a lot of control over the important things in my life | .924 |
| I feel safe where I live | .924 |
| The local shops, services and facilities are good overall | .925 |
| I get pleasure from my home | .925 |
| I find my neighbourhood friendly | .925 |
| I take life as it comes and make the best of things | .923 |
| I feel lucky compared to most people | .924 |
| I tend to look on the bright side | .925 |
| If my health limits social/ leisure activities, then I will compensate and find something else I can do | .925 |
| I have enough money to pay for household bills | .926 |
| I have enough money to pay for household repairs or help needed in the house | .926 |
| I can afford to buy what I want to | .925 |
| I cannot afford to do things I would enjoy | .928 |
| I have social or leisure activities/hobbies that I enjoy doing | .924 |
| I try to stay involved with things | .926 |
| I do paid or unpaid work or activities that give me disagree a role in life | .926 |
| I have responsibilities to others that restrict my social or leisure activities | .925 |
| Religion, belief or philosophy is important to my quality of life | .926 |
| Cultural/religious events/festivals are important to my quality of life | .926 |
